# When the Brain's Switchboard Short Circuits: Cell‐Type Specific Molecular Characterization of the Human Thalamus in Alzheimer's Disease

**DOI:** 10.1002/alz70855_105194

**Published:** 2025-12-24

**Authors:** Rio McLellan, Laura Kus, David A. Davis, Thomas S Carroll, Christina Pressl, Ester Siantoputri, Nathaniel Heintz

**Affiliations:** ^1^ Rockefeller University, New York, NY, USA; ^2^ The Rockefeller University, New York City, New York, NY, USA; ^3^ Miller School of Medicine, The University of Miami, Miami, FL, USA; ^4^ Bioinformatics Resource Center, The Rockefeller University, New York, NY, USA

## Abstract

**Background:**

Selective cellular vulnerability is a hallmark of several late onset neurodegenerative disorders, including Alzheimer's Disease (AD). In AD, neurofibrillary tangles first accumulate in the transentorhinal cortex, subsequently spreading to the hippocampus and across the cerebrum. Consequently, existing research has primarily focused on these regions. However, other brain areas affected early in AD, such as the thalamus, remain underexplored. While the thalamus is well‐known as the brain's primary sensorimotor relay station, it's increasingly recognized as playing a crucial role in memory and spatial navigation. Postmortem studies reveal widespread amyloid and tau deposition across various thalamic nuclei early in the pathophysiology of AD. Furthermore, neuroimaging studies demonstrate significant thalamic atrophy, white matter alterations, and functional connectivity changes to occur in the initial stages of cognitive decline, prior to AD conversion. Despite these observations, little is known about selective cellular vulnerability within the thalamus and its contribution to cognitive and functional decline in AD.

**Method:**

Immunohistochemical neuropathological profiling was used to identify vulnerable thalamic nuclei in postmortem brain tissue from early AD donors. These regions were sub‐dissected, and nuclei were isolated from human control (Braak Stage 0) and early AD (Braak Stages I‐IV) postmortem brain tissues using established protocols. Fluorescence‐activated nuclear sorting (FANS) was employed to isolate specific thalamic cell types for bulk RNA sequencing. Analysis of gene expression profiles for each cell type confirmed the effectiveness of this approach in selectively and reproducibly isolating thalamic cell populations.

**Result:**

Using FANS, we successfully isolated six distinct thalamic cell types from AD‐implicated nuclei in postmortem human brain tissue from both control and early AD donors. These cell types include glutamatergic thalamic projection neurons (TPNs), GABAergic inhibitory interneurons, astrocytes, microglia, oligodendrocytes, and oligodendrocyte precursor cells (OPCs). We will next perform comprehensive transcriptional and epigenetic profiling to investigate cell‐type specific changes in the human thalamus during early AD.

**Conclusion:**

Neuropathological profiling of early AD donors supports previous studies indicating that the thalamus is affected early in AD. Our preliminary results indicate that our current dissection and sorting strategy will be valuable for uncovering AD‐related alterations in the thalamus and elucidating its role in disease progression.

